# 5-Aza-2′-Deoxycytidine Regulates White Adipocyte Browning by Modulating miRNA-133a/Prdm16

**DOI:** 10.3390/metabo12111131

**Published:** 2022-11-17

**Authors:** Jia Liang, Ying Jia, Huixin Yu, Haijing Yan, Qingyu Shen, Yong Xu, Yana Li, Meizi Yang

**Affiliations:** 1 Department of Pharmacology, Binzhou Medical University, Yantai 264003, China; 2 Department of Pathophysiology, Binzhou Medical University, Yantai 264003, China

**Keywords:** 5-Aza-dC, white adipocytes, brown adipocytes, Prdm16, Ucp-1

## Abstract

The conversion of white adipocytes into brown adipocytes improves their thermogenesis and promotes energy consumption. Epigenetic modifications affect related genes and interfere with energy metabolism, and these are the basis of new ideas for obesity treatment. Neonatal mice show high levels of DNA hypermethylation in white adipose tissue early in life and low levels in brown adipose tissue. Thus, we considered that the regulation of DNA methylation may play a role in the conversion of white adipose to brown. We observed growth indicators, lipid droplets of adipocytes, brown fat specific protein, and miRNA-133a after treatment with 5-Aza-2′-deoxycytidine. The expression of Prdm16 and Ucp-1 in adipocytes was detected after inhibiting miRNA-133a. The results showed a decrease in total lipid droplet formation and an increased expression of the brown fat specific proteins Prdm16 and Ucp-1. This study indicated that 5-Aza-2′-deoxycytidine promotes white adipocyte browning following DNA demethylation, possibly via the modulation of miR-133a and Prdm16.

## 1. Introduction

The development of obesity and obesity resistance is closely linked to the energy metabolism of adipose tissue. Mammalian adipose tissues are typically divided into white adipose tissue (WAT), brown adipose tissue (BAT) and beige adipocytes [1]. White adipocytes in rodent subcutaneous fat change to beige adipocytes when stimulated by cold. The number and the size of the mitochondria were significantly increased in the cells containing small lipid droplets with multiple compartments [2]. WAT allows the body to quickly replenish energy when needed, but the excessive accumulation of WAT can lead to obesity [3,4,5]. Brown adipocytes are characterized by densely packed mitochondria, which generate heat and consume energy through the combustion of coupling protein 1 (Ucp-1) in the mitochondria [6,7,8]. White adipocytes can be converted to brown adipocytes under the influence of some regulatory factors.

Obesity is the result of the interaction between environmental and genetic factors, with epigenetics as a logical interface between genes and the environment. DNA methylation is one of the most important epigenetic mechanisms regulating gene expression [9]. It affects the differentiation of adipocytes and energy metabolism. Studies have found an overall hypermethylation of mature adipocytes compared to preadipocytes, as well as in white adipocytes compared to brown adipocytes [10,11]. This suggests that the early methylation inhibition of adipocyte formation may be a potential approach to promoting the conversion of white adipocytes to brown adipocytes. The methyltransferase-specific inhibitor 5-Aza-2-deoxycytidine (5-Aza-dC) is a pyrimidine analogue which reverses DNA methylation. In recent years, studies have suggested that the inhibition of DNA methylation by 5-Aza-dC leads to a lower lipid droplet accumulation in 3T3-L1 at the early differentiation stage and suppresses adipogenesis [12,13,14]. Therefore, we consider that the inhibition of DNA methylation may affect white fat browning. 

There is a close inter-regulatory relationship between different epigenetic modifications, which in turn affect the expression of downstream target genes and cause changes in the organism. MicroRNAs are involved in the differentiation of adipose tissue and play an important direct regulatory role in white fat browning [15]. While the inhibition of miR-133a increases brown adipocyte gene expression, miR-133 directly targets the 3′UTR of the Prdm16 mRNA to repress Prdm16 expression [16,17,18]. These findings suggest that miR-133a may be a possible strategy for treating obesity. The regulatory role of miRNAs in browning occurs through transcription factors, including the positive regulatory domain zinc finger protein16 (Prdm16) and the peroxisome proliferator activated receptor-γ (PPARγ) [19]. Prdm16 is a zinc finger protein which is important for the formation of brown fat and is distributed in various organs and tissues, such as the heart, liver, kidney, brain, embryo, skeletal muscle, adipose tissue, etc. [20]. Mechanistic studies have shown that the knockdown of Prdm16 disrupts the thermogenic properties of brown adipocytes and also causes an increase in white adipose-specific gene expression [21,22]. These studies strongly suggest that Prdm16 is a key factor in brown adipocyte differentiation. Prdm16 induces a brown adipose phenotype by activating PGC-1a and Ucp-1 [5,6,10]. Ucp-1, the key thermogenic factor, which is unique to brown adipocytes, uncouples the oxidation of ATP-generated substrates to generate heat. Its high specificity of expression in brown adipose tissue makes it an important indicator of brown adipocyte formation and activity [8,23,24]. Its importance in brown adipocyte differentiation prompted us to consider Prdm16 and Ucp-1 as key proteins in this study.

In this study, we used a DNMT1 enzyme inhibitor (5-Aza-dC) to inhibit the extent of DNA methylation early in the life of neonatal C57BL/6J mice in order to investigate the effect of 5-Aza-dC on white fat browning. We aimed to explore the role and the possible mechanisms of the altered DNA methylation early in life with respect to white fat browning and provide an important basis for the future research on obesity.

## 2. Materials and Methods

### 2.1. Animals 

C57BL/6J mice (No. SCXK2019-0003; Jinan Peng Yue Experimental Animal Breeding Co., Ltd., Jinan, China) were used to establish the obesity models. All the animal experiments were conducted in compliance with the National Institutes of Health Guide for the Care and Use of Laboratory Animals and approved by the Binzhou Medical University Animal Experimentation Committee (approval No. 2017002). 

**Figure 1:**
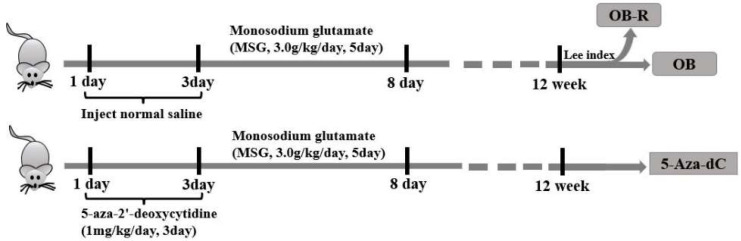
Graphic of the animal model design.

The mice were randomly divided into three groups: the normal group, obesity group (OB), and 5-Aza-dC group. In the obesity group, newborn mice were subcutaneously injected with an equal dose of saline from day 1 after birth for 3 consecutive days and MSG from day 4 after birth for 5 consecutive days. At 12 weeks of age, the mice were divided into the OB group and MSG-induced obesity-resistant group (OB-R). The 5-Aza-dC group was subcutaneously injected with an equal dose of 5-aza-2′-deoxycytidine (5-Aza,1 mg/kg/d; Sigma-Aldrich, St. Louis, MO, USA) from day 1 after birth for 3 consecutive days and monosodium glutamate (MSG, 3.0 g/kg; Sigma-Aldrich, St. Louis, MO, USA) from day 4 after birth for 5 consecutive days. The mice were weaned 3 weeks after birth and raised in stainless steel cages (Figure 1). All the mice were kept under a 12/12 h light/dark cycle under ventilated, dry conditions at 22 ± 2 °C and had ad libitum access to food and water. 

Timeline showing the animal model. All mice were sacrificed at 12 weeks, and tissues were harvested for analysis. OB, MSG-induced obese mice; OB-R, MSG-induced obesity-resistant mice; 5-Aza-dC,5-aza-2′-deoxycytidine-induced DNA demethylation mice.

### 2.2. Determination of Parameters

Each mouse was weighed with an electronic balance (Mettler Toledo, Columbus, OH, USA), and body temperature was measured behind the ear with an infrared thermometer (GUIDE, Wuhan, China). A ruler was used to measure the nose-to-anus body length, and the average food intake was measured three times weekly. The Lee index was calculated as follows: body weight (g)1/3 × 103/length (cm).

Perirenal WAT, inguinal subcutaneous WAT, gonadal WAT, and interscapular BAT were obtained from all mice, which were dissected, weighed and recorded, and stored at −80 °C for further study.

### 2.3. Cells

Adipose tissue was taken from the ventral groove region of the animals and rinsed with PBS, and adipose tissue was sheared and centrifuged. The upper adipose tissue was taken, digested with 0.1% type I collagenase (Sigma-Aldrich, St. Louis, MO, USA) in a constant temperature incubator for 1 h, lysed for 2 min with red blood cell lysate after filtration, and centrifuged. The lysis was terminated with PBS. After centrifugation, complete medium was added to the pellet and filtered again, and the adipocytes were collected for the culture. The primary adipocytes were cultured in Dulbecco’s modified Eagle’s medium mixture F-12 (DMEM F-12, HyClone, Logan, UT, USA), supplemented with 10% fetal bovine serum (FBS, Thermo Fisher Scientific, Waltham, MA, USA) at 37 °C in a humidified atmosphere with 5% CO_2_ until day 8. The medium was changed to a drug medium (DMEMF-12 containing 10%FBS, 5 µM 5-azacytidine), and the samples were placed in a constant temperature incubator at 37 °C and 5% CO_2_ for 48 h. Then, the complete medium was replaced without 5-Aza, followed by further incubation in the incubator for 48 h and subsequent photography, oil red O staining, or further study.

### 2.4. Oil Red O Staining

On day 12, the cells were washed in phosphate-buffered saline (PBS), fixed with freshly prepared 4% formaldehyde for 30 min, and stained using Oil Red O working solution (Titan, Shanghai, China) for 30 min. The cells were washed in PBS.

### 2.5. Determination of 5-Methyl Cytosine in Adipose Tissue

The DNA methylation product 5-methyl cytosine was measured using a mouse 5-methyl cytosine ELISA kit (Enzyme-linked Biotech, Shanghai, China), and the optical density of the samples was analyzed at 450 nm using an enzyme standard instrument (Bio-Tek, Winooski, VT, USA). 

### 2.6. Determination of Serum Leptin Levels

Serum leptin was measured using a mouse leptin ELISA kit (Enzyme-linked Biotech, Shanghai, China), and the optical density of the samples was analyzed at 450 nm using an enzyme standard instrument (Bio-Tek, Winooski, VT, USA).

### 2.7. Histology and Immunofluorescence

WAT is extracted from animals, fixed, embedded, and cut into 4 mm-thick slices. The slides were stained with H&E and measured under a microscope. Immunofluorescence staining was performed according to the standard protocol, using the following antibodies: Prdm-16 (Affinity, rabbit, 1:200) and Ucp-1 (R&D, mouse, 1:50). The samples were first permeabilized with 0.3% Triton X-100, blocked with 5% goat serum solution, and incubated with the primary antibody overnight in a humidified chamber at 4 °C. After washing three times, the samples were incubated for 2 h at 37 °C with secondary antibodies conjugated to FITC (1:200, Thermo Fisher Scientific, Waltham, MA, USA) and CY3 (1:200; Abbkine, San Diego, CA, USA). They were then counterstained with 4′,6-diamidino-2-phenyl-indole (DAPI) for the staining of the nuclei.

The cells were washed in PBS and fixed with freshly prepared 4% formaldehyde, and immunofluorescence assays were performed as described above.

### 2.8. Western Blotting

The extracted adipose tissue and cells were lysed and centrifuged to obtain the supernatant. After protein extraction, protein concentrations were determined using a BCA kit (Thermo Fisher Scientific, Waltham, MA, USA), and proteins were separated on 8% sodium dodecyl thiodifluoride-polyacrylamide gels and transferred to polyvinylidene difluoride membranes. Block and then use anti-PRDM16 (1:1000; Affinity, Cincinnati, OH, USA), anti-UCP-1 (1:1000, affinity), and anti-glyceraldehyde 3-phosphate dehydrogenase (GAPDH; 1:1000 affinity) antibody at 4 °C overnight. After washing, the horseradish peroxidase-conjugated IgG was incubated for 2 h. Bands were detected by ImageJ software (v.1.37; NIH, Bethesda, MD, USA) for grayscale analysis. The results are shown as the ratio of the intensity of the target protein to the GAPDH band.

### 2.9. RT-PCR Analysis

Total RNA was extracted using the TRIzol method (Invitrogen, Waltham, MA, USA) in adipose tissue and cells. The RT reaction was carried out using 2 μg of total RNA, the RNA was reverse-transcribed into complementary DNA using a reverse transcription kit (Gene Pharma, Shanghai, China). According to the manufacturer’s instruction, quantitative real-time polymerase chain reaction (qRT-PCR) was performed on a Real Time PCR system (Bio-Rad, Hercules, CA, USA) using the Hairpin-it TM microRNA and U6 snRNA Normalization RT-PCR Quantitation kit (Gene Pharma). Using U6 as a control, the relative gene expression was quantitatively normalized using the 2−ΔΔCt analysis method to detect microRNA expression. Primers were designed as follows: mmu-miR-133a, F: 5′-GCCTTTGGTCCCCTTCAAC-3′, R: 5′-TATGCTTGTTCTCGTCTCTGTGTC-3′; rno-miR-133a, F: 5′-GCCTTTGGTCCCCTTCAAC-3′, R: 5′-TATGCTTGTTCTCGTCTCTGTGTC-3′; U6 snRNA F: 5′-CAGCACATATACTAAAATTGGAACG-3′, R: 5′-ACGAATTTGCGTGTCATCC-3′.

### 2.10. MicroRNA Transfection of Primary Adipocytes

According to the manufacturer’s instructions, the miR133a inhibitor or mimic (Gene Pharma, Shanghai, China) was transfected into mature adipocytes cultured in serum-free medium using Lipofectamine^®^ RNAi MAX Reagent (Thermo Fisher Scientific, Waltham, MA, USA). After 6 h, we replaced the old medium with DMEM complete medium containing 10% FBS and incubated the cells at 37 °C for 1–3 days. We analyzed the transfected cells according to the specific situation. Primers were designed as follows: miR-133a inhibitor, 5′-CAGCUGGUUGAAGGGGACCAAA-3′; NC inhibitor 5′-CAGUACUUUUGUGUAGUACAA-3′.

### 2.11. Statistical Analysis

All the values are shown as a one-way and two-way analysis of variance with Dunnett’s T3 post hoc tests and were obtained using the GraphPad Prism software (v.8.0.1; GraphPad Software, La Jolla, CA, USA). All the results were considered significant at *p* < 0.05.

## 3. Results

### 3.1. DNA Methyltransferase Inhibitor 5-Aza-2′-Deoxycytidine Influenced Normal Growth Indicators in Mice

**Figure 2:**
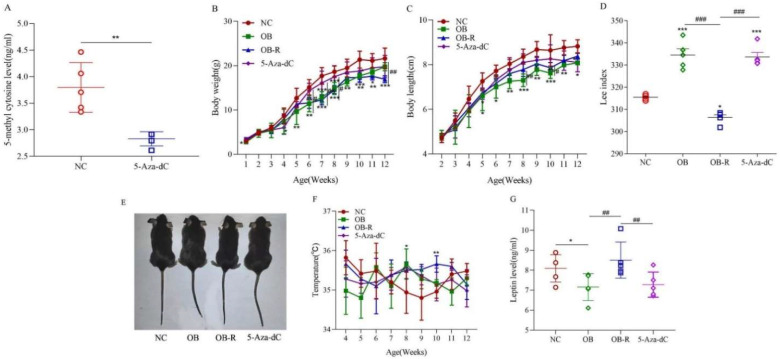
The effect of 5-Aza-dC on the growth characteristics of mice. (**A**) Identification of 5-methyl cytosine levels after 5-Aza-dC treatment, presented as the mean ± SD (n = 5). (**B**) Body weights in the NC, OB, OB-R, and 5-Aza-dC groups from weeks 1 to 12 are presented as the mean ± SD (n = 5). (**C**) Body lengths of mice in the four groups from weeks 2 to 12 are presented as the mean ± SD (n = 5). (**D**) Lee indices in the four groups at week 12 are presented as the mean ± SD (n = 5). (**E**) Four mice from the same litter; the first shows the general appearance of a normal mouse, the second shows an obese mouse, the third shows an obesity-resistant mouse, and the fourth shows a 5-aza-2′-deoxycytidine-induced mouse. (**F**) Body temperatures in the three groups from weeks 4 to 12 are shown as the mean ± SD (n = 5). (**G**) Levels of the four groups’ leptin levels in mouse serum are presented as the mean ± SD (n = 5). * *p* < 0.05; ** *p* < 0.01; *** *p* < 0.001 vs. the NC group; # *p* < 0.05; ## *p* < 0.01; ### *p* < 0.001, compared with each other. NC, normal control; OB, MSG-induced obese mice; OB-R, MSG-induced obesity-resistant mice; 5-Aza-dC, 5-aza-2′-deoxycytidine-induced mice.

The mice were treated with DNA methyltransferase inhibitor 5-aza-2′-deoxycytidine (5-aza) from the day of birth, while the same dose of MSG reagent as that given to the obese group was given on day 4. As shown in Figure 2A, after 2 weeks of drug treatment, the modeling results were identified by measuring the level of DNA methylation product 5-methyl cytosine (5-methyl cytosine), and the results showed that the methylation level of 5-Aza-dC-treated mice was significantly lower than that of the NC group (*p* < 0.01). 

The growth indicators of the mice were recorded and analyzed after 5-Aza-dC treatment. At 5 weeks of age, the body weights of the mice in the 5-Aza-dC group were higher than those in the OB group and the OB-R group and lower than those in the NC group. Similarly, the body lengths of the mice in the 5-Aza-dC group were higher than those in the OB group and the OB-R group and lower than those in the NC group (*p* < 0.001; *p* < 0.01; *p* < 0.05; Figure 2B,C). At the end of the experiment (12 weeks), based on the weight and body length data, the Lee index of the 5-Aza-dC group was not significantly different from that of the OB group (*p* > 0.05) but was significantly higher than that of the OB-R group and the NC group (*p* < 0.001; Figure 2D). As shown in Figure 2E, 5-Aza-dC-induced mice did not have the body shape of obese mice, with a shortened body length and fat accumulation, although their Lee index did not differ from that of obese mice. Although body temperature did not differ significantly between groups, it was generally higher in the 5-Aza-dC group and showed smaller fluctuations (*p* > 0.05; Figure 2F). As shown in Figure 2G, the leptin levels in the 5-Aza-dC groups were slightly lower than those in the OB-R group and NC group and were slightly higher than those in the OB group, although there was no significant difference (*p* > 0.05). Preliminary growth index data indicate that 5-Aza-dC-treated mice grow normally and have growth characteristics similar to those of obese mice, but with differences in body size. 

### 3.2. Adipose Tissue of Mice Exhibits Browning Characteristics after 5-Aza-dC Treatment

**Figure 3:**
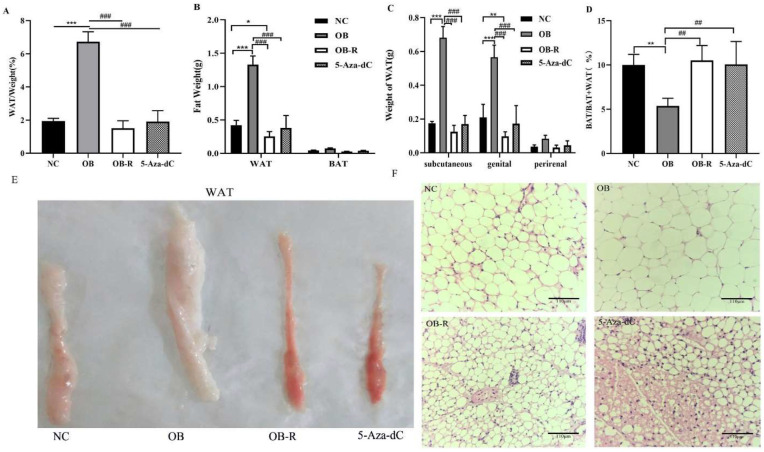
Detection of the subcutaneous adipose tissue in mice after 5-Aza-dC treatment. (**A**) Body fat percentages of the four groups are presented as the mean ± SD (n = 5); (**B**) Fat weights of mice in the four groups are presented as the mean ± SD (n = 5); (**C**) WAT distribution in mice in the four groups is presented as the mean ± SD (n = 5); (**D**) The proportion of brown fat weight to total fat weight in the four groups, presented as the mean ± SD (n = 5). (**E**) Pictures of subcutaneous white adipose tissue of the four groups; (**F**) H&E staining in the inguinal subcutaneous WAT of mice induced by MSG (scale bar = 110 μm). * *p* < 0.05; ** *p* < 0.01; *** *p* < 0.001 vs. the NC group; ## *p* < 0.01; ### *p* < 0.001 vs. each other group. NC, normal control; OB, MSG-induced obese mice; OB-R, MSG-induced obesity-resistant mice; 5-Aza-dC, 5-aza-2′-deoxycytidine-induced DNA demethylation mice; WAT, white adipose tissue; BAT, brown adipose tissue.

Because the weight of adipose tissue allows for a further assessment of obesity characteristics, we performed a comparative analysis of WAT and BAT in each group. First, we analyzed the proportion of white adipose tissue to mouse body weight from the four groups and initially confirmed that the body fat percentage of mice in the 5-Aza-dC group was significantly lower than that of mice in the OB group but not statistically significant compared to the OB-R group (*p* < 0.001; Figure 3A). In addition, we also analyzed the absolute tissue weights of WAT and BAT in the four groups and found that WAT was significantly lower in the 5-Aza-dC group than it was in the OB group, though there was no significant difference compared to the OB-R group (*p* < 0.001; *p* > 0.05; Figure 3B). The BAT was not significantly different among the four groups; however, as with the OB-R group, the proportion of BAT weight to total fat weight in the 5-Aza-dC group was significantly higher than that of the OB group (*p* < 0.01; Figure 3D). The WAT in various parts of the body differed in these groups; the WAT weight distribution in each part of the 5-Aza-dC group was always lower than that in the OB group (*p* < 0.001; Figure 3C). In summary, although the total fat weight of mice decreased after DNA demethylation, the proportion of brown fat increased significantly, showing that 5-Aza-dC affected the development of adipose tissue and increased the proportion of brown fat. 

White and brown adipocytes show different morphologies and functions. White adipocytes are single-compartment, large lipid droplets, while brown adipocytes are multi-compartment, small lipid droplets. There are data suggesting that the subcutaneous white fat in mice is more susceptible to browning compared to visceral white fat [25]. We observed the subcutaneous white adipose tissue in each group of mice and performed HE staining. As shown in Figure 3E,F, compared to the OB group, the subcutaneous WAT in the 5-Aza-dC group and OB-R groups had brown adipose tissue-like features under H&E staining. Lipid droplets become smaller and appear to resemble brown adipocytes, which is a manifestation of WAT browning, with the adipose tissue in the OB group having more of a white adipose character (Figure 3E,F). In summary, we morphologically observed the subcutaneous inguinal white adipose tissue in mice, and the results further demonstrated that the 5-Aza-dC inhibition of DNA methylation affected the conversion between subcutaneous adipose tissues in mice and subcutaneous WAT exhibiting the characteristics of white fat browning. 

### 3.3. 5-Aza-dC Promotes the Browning of WAT by Regulating Related Factors In Vivo

**Figure 4:**
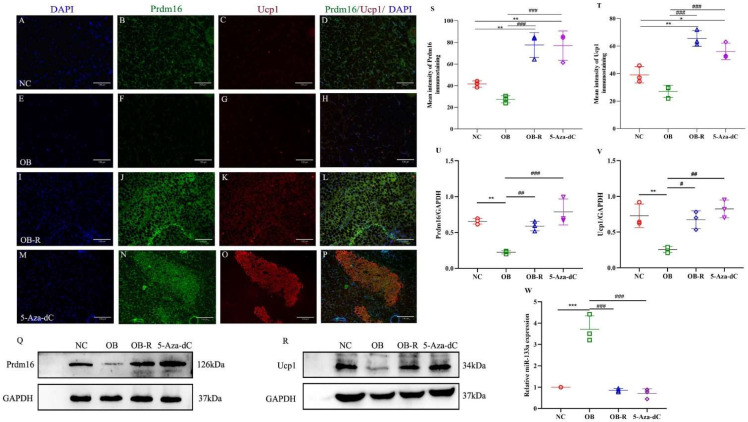
Prdm16, Ucp-1, and miR-133a expression in subcutaneous WAT. (**A**–**P**) Increased immunoreactivity of Prdm16 (green) and Ucp1 (red) after DNA demethylation (n = 3/group; blue, DAPI). (**Q**,**R**) Expression of Prdm16 and Ucp1 in subcutaneous WAT using Western blotting. (**S**,**T**) Quantified changes in Prdm16 and Ucp1 after DNA demethylation (n = 3/group). (**U**,**V**) Normalized intensity of Prdm16 and Ucp1 relative to GAPDH, presented as the mean ± SD (n= 3/group). (**W**) The expression levels of miR-133a in the subcutaneous WAT of the four groups (n= 3/group). *** *p* < 0.001; ** *p* < 0.01; * *p* < 0.05, compared with controls; # *p* < 0.05; ## *p* < 0.001; ### *p* < 0.001, compared with each other. NC, normal control; OB, MSG-induced obese mice; OB-R, MSG-induced obesity-resistant mice; 5-Aza-dC, 5-aza-2′-deoxycytidine-induced DNA demethylation mice; Prdm16, PR domain containing 16; Ucp-1, uncoupling protein-1; WAT, white adipose tissue.

Considering that Prdm16 plays a key role in the development of brown adipocytes and Ucp1 is an important marker of brown adipocytes, we assessed the expression levels of brown adipose marker genes. The level of browning marker proteins (Prdm16/Ucp1) in adipose tissue was assessed using immunofluorescence and Western blotting (Figure 4A–R). The results showed that Prdm16 and Ucp1 protein expression was significantly higher in the 5-Aza-dC and OB-R groups than it was in the OB group, and the protein expression levels were lowest in the subcutaneous WAT of mice in the OB group (*p* < 0.001; *p* < 0.01; *p* < 0.05; Figure 4S–V). 

There is a close interrelationship between epigenetic regulation and DNA methylation, which can affect the expression activity of downstream target genes through the regulation of miRNAs. Prdm16 is known to be a direct target of miR-133a, and the expression of miR-133a was assessed using RT-PCR. The expression level of miRNA-133a in the 5-Aza-dC group was the lowest, and the OB group had the highest expression level (*p* < 0.001; Figure 4W). The results showed that mice treated with 5-Aza-dC exhibit the browning of white fat, which may be promoted by the downregulation of miR-133a. 

### 3.4. 5-Aza-dC Inhibited the Accumulation of Lipid Droplets and Promoted the Browning of Adipocytes

**Figure 5:**
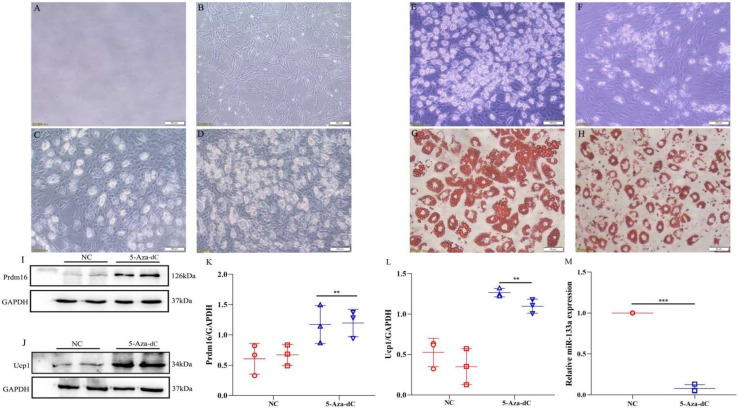
Detection of Prdm16, Ucp-1, and miR-133a expression in adipocytes (**A**–**D**). Primary adipocyte culture for the 0–12th day (**A**–**D**, Bar = 100 µm). (**E**) The growth of cells on the 12th day after DNA demethylation treatment; (**F**) The growth of cells on the 12th day (**E**,**F**, Bar = 100 µm); (**G**) Oil red staining of cells on the 12th day after DNA demethylation treatment; (**H**) Oil red staining of cells on the 12th day (**G**,**H**, Bar = 50 µm). (**I**,**J**) Expression of Prdm16 and Ucp1 in adipocytes using Western blotting. (**K**,**L**) Normalized intensity of Prdm16 and Ucp1 relative to GAPDH is presented as the mean ± SD (n= 3). (**M**) The expression levels of miR-133a in fully differentiated adipocytes treated with 5-aza. *** *p* < 0.001, ** *p* < 0.01, compared with controls. NC, normal control; 5-Aza-dC, 5-aza-2′-deoxycytidine-induced adipocytes.

We extracted and cultured primary adipocytes, observed the adipocyte morphology, and found that the cells differentiated into mature adipocytes from the 8th day to the 12th day (Figure 5A–D). Oil Red O staining showed that the mature adipocytes under 5-aza-2′-deoxycytidine treatment contained significantly fewer lipid droplets than control cells, and the fat droplets in the control cells were larger than those in the 5-aza-treated cells (Figure 5E–H). The results suggested that DNA demethylation may inhibit the differentiation of adipocytes and the accumulation of lipid droplets.

We further assessed the effect of DNA demethylation on brown fat-specific associated proteins in adipocytes and examined the expression of Prdm16 and Ucp1 by Western blot (Figure 5I,J). Consistent with our findings in adipose tissue, the expression levels of miR-133a were downregulated with 5-aza-2′-deoxycytidine treatment in adipocytes to induce Prdm16 and Ucp1 expression. Western blot analysis also showed an increase in the protein levels of Prdm16 and Ucp1 (*p* < 0.01; Figure 5K–M). These results suggested that DNA demethylation induces the expression of brown fat marker genes in adipocytes, accompanied by changes in lipid droplet accumulation. 

**Figure 6:**
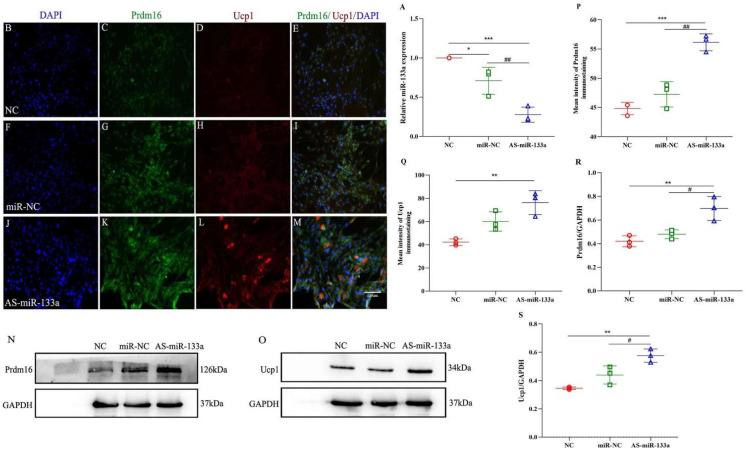
Immunoreactivity of Prdm16/Ucp1 proteins after the inhibition of miR-133a expression. (**A**) The expression levels of miR-133a in fully differentiated adipocytes treated with 5-aza and transfected with or without miR-133a inhibitor. (**B**–**M**) Increased immunoreactivity of Prdm16 (green) and Ucp1 (red) in fully differentiated adipocytes treated with 5-aza and transfected with or without miR-133a inhibitor (n = 3/group; blue, DAPI). (**N**,**O**) Expression of Prdm16 and Ucp1 in fully differentiated adipocytes treated with 5-aza and transfected with or without miR-133a inhibitor using Western blotting. (**P**,**Q**) Quantified changes in Prdm16 and Ucp1 in fully differentiated adipocytes treated with 5-aza and transfected with or without miR-133a inhibitor. (**R**,**S**) Normalized intensity of Prdm16 and Ucp1 relative to GAPDH, presented as the mean ± SD (n= 3). * *p* < 0.05, ** *p* < 0.01, *** *p* < 0.001 compared with the control group; # *p* < 0.05, ## *p* < 0.01 compared with other groups; bar = 130 μm. Prdm16, PR domain containing 16; Ucp-1, uncoupling protein-1; miR-133a, microRNA-133a; 5-aza-dC, 5-aza-2′-deoxycytidine.

**Figure 7:**
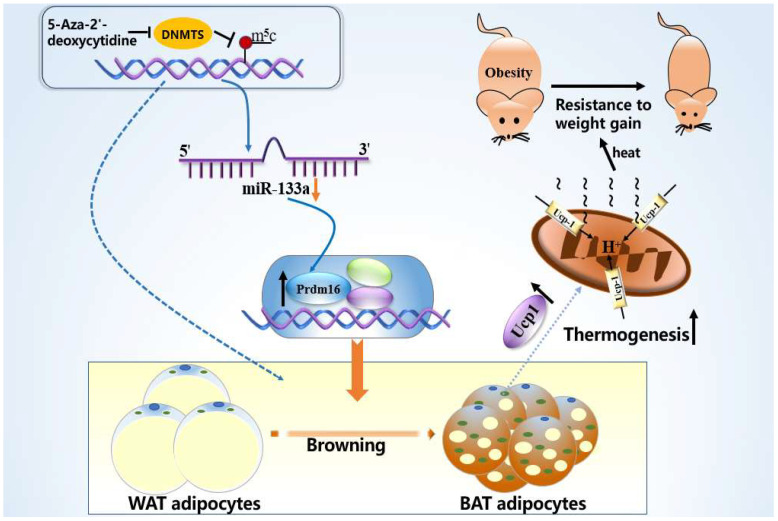
Graphic of 5-Aza-dC-mediated browning of white adipocytes. 5-Aza-dC suppresses the expression of miR-133a, which is a direct inhibitor of Prdm16. Thus, the 5-Aza-dC-mediated suppression of miR-133a upregulates the expression of Prdm16 and promotes the browning of white fat, increasing Ucp-1 expression and thermogenic activity, which contributes to resistance to weight gain. miR-133a, microRNA-133a; Prdm16, PR domain containing 16; Ucp-1, uncoupling protein 1.

To further determine the involvement of miR-133a in 5-aza-mediated browning, we transfected an miR-133a inhibitor into adipocytes and reduced its level, which was further lowered by 5-Aza (*p* < 0.001; Figure 6A). Furthermore, we simultaneously boosted the expression of brown fat marker proteins, as shown by immunofluorescence and Western blotting, including Prdm16 and Ucp1, compared to 5-Aza-dC only (*p* < 0.001; *p* < 0.01; *p* < 0.05; Figure 6B–S). These data support the effect of miR-133a depletion on Prdm16 regulation, and through these data, we found that 5-Aza-dC regulates the brown marker protein Prdm16 by affecting the expression of miR-133a, which promotes the browning of white fat and increases the expression of the thermogenic protein Ucp-1 (Figure 7).

## 4. Discussion

Obesity is the result of energy intake chronically exceeding energy expenditure and is closely associated with the onset and development of metabolic diseases. Research data have shown that obese mice induced by high fat levels in the same environment have two models of obesity and obesity resistance. The weight of the obesity-resistant model was significantly lower than that of the obesity model. It has been suggested that the obesity-resistant phenomenon might be related to metabolic genes [26,27]. The researchers also found that C57BL/6 mice were more prone to obesity, fat accumulation, and altered adipocyte sizes than other strains of mice. Therefore, C57BL/6 mice are widely used in metabolic disease research, especially for obesity models. Consistent with previous results, the MSG-induced obese mice in our study did not gain weight significantly and had a shorter body length and fat accumulation. Hypothalamic neuropeptides contain distinct nuclei and form a complex regulatory network that regulates dietary behavior and energy balance. The obesity model was induced by affecting the hypothalamus to interfere with the feeding center [28].

It is well known that the excessive accumulation of white adipose tissue causes obesity, while brown adipose tissue dissipates heat by burning fat, and the higher the proportion of BAT, the more heat production occurs. Therefore, obesity can be solved by increasing the activity of BAT or inducing BAT-like cells to promote energy consumption [29]. Increasingly, researchers are using white fat browning to increase energy consumption to address obesity, opening up a new anti-obesity treatment direction. DNA methylation, one of the most widely studied epigenetic mechanisms, has been shown to play a key role in regulating adipose cell differentiation [30]. In our study, DNA methyltransferase inhibitors were used to inhibit the methylation level of mice. Based on the data of body weight and body length, the Lee index of DNA demethylated mice was not significantly different from that of the obese group, but it was significantly higher than that of the obesity-resistant group. The growth index of the mice reflected that DNA methylation affected fat metabolism but did not affect normal growth. Some researchers have comprehensively analyzed the DNA methylome of brown and white adipocytes during the course of differentiation, revealing differential methylation between white and brown adipocytes during cell differentiation [11]. Although the Lee index was the same as that of obese mice, the weight of the adipose tissue of mice treated with 5-Aza-dC was much lower than that of the obese group, while the proportion of BAT to total fat weight was significantly higher than that of the obese group, indicating that the weight of adipose tissue decreased and the proportion of brown fat increased significantly. In fact, there is no positive correlation between body weight and body fat percentage. Brown fat cells and skeletal muscle cells originate from common precursor cells, and the oxidative metabolism, color, and mitochondrial content of brown fat are similar to those of skeletal muscle. In addition, epigenetic regulation is crucial to skeletal muscle development, and DNA methylation is also a key mechanism driving muscle development [31,32,33,34]. Therefore, the inhibition of DNA methylation may cause a partial conversion of adipocytes to skeletal muscle; however, the exact mechanism of its formation is still unclear, and further research is needed. 

The increase in the Lee index was accompanied by a decrease in the weight of adipose tissue, which prompted us to further detect the characteristics of white fat in mice. We found that 5-Aza-dC-treated mice showed the same color as obese-resistant mice with white adipose tissue; the color of the adipose tissue appeared brown, and in the microscopic observation of the adipose tissue morphology, the lipid droplet size was significantly reduced. We also noted increased expression of the brown adipocyte thermogenic gene uncoupling protein-1 (Ucp-1) and the transcriptional regulator Prdm16 protein, which controls classical brown adipocyte development in mice [20,21,35]. Prdm16 enhances the Ucp-1-mediated thermogenic gene programming in subcutaneous white adipose, promoting energy expenditure and improving metabolism, which resists obesity [36]. Consistent with the trend of the morphological results, the brown adipose-specific associated protein Prdm16 was significantly increased in the subcutaneous white adipose tissue by 5-Aza-dC, along with a simultaneous increase in the brown adipose-specific thermogenic protein Ucp1. Thus, 5-Aza-dC may have increased the expression of brown adipose-specific proteins, which in turn promoted the browning of white adipose in mice. MicroRNAs play important roles in both brown fat development and the browning of white adipocytes. 5-Aza-dC alters epigenetic modifications by inhibiting DNA methylation, which interacts with microRNA, and miR-133a plays an important role in the browning of white fat [37,38,39]. The original lipid droplets of the adipocytes were significantly reduced by 5-Aza-dC in primary adipocytes, and the miR-133a expression levels decreased. Therefore, it is speculated that the effect of 5-Aza-dC on the browning of white fat by inhibiting DNA methylation may be related to the regulation of miR-133a and Prdm16. However, the inhibition of miR-133a significantly increased Prdm16 and Ucp1; thus, 5-Aza may promote the browning of white fat through the miR-133a/Prdm16 pathway, and it also increases the expression of the thermogenic protein Ucp1, which generates heat.

In summary, the morphology and related proteins of white fat in mice showed browning characteristics with DNA methyltransferase inhibitor 5-Aza-dC, which affected miR-133a expression through epigenetic regulation and acted on the browning marker protein Prdm16, resulting in adipocyte browning, which in turn may act as a resistance to obesity. This result provides a new theoretical method for adipocyte browning in mice and may provide a feasible target for obesity research.

## Data Availability

The data presented in this study are available in this article.
